# P-1245. An Open-Label Phase 1 Study to Evaluate the Effect of Multiple Doses of Rifabutin on the Single-Dose Pharmacokinetics of Ulonivirine in Adults without HIV

**DOI:** 10.1093/ofid/ofaf695.1437

**Published:** 2026-01-11

**Authors:** Makeda Robinson, Randolph P Matthews, Bhargava Kandala, Akshita Chawla, Arinjita Bhattacharyya, Carol Cannon, Andrea Schaeffer, Allen Hunt, S Aubrey Stoch, Jesse C Nussbaum

**Affiliations:** Merck & Co., Inc., South San Francisco, CA; Merck & Co., Inc., South San Francisco, CA; Merck & Co., Inc., South San Francisco, CA; Merck & Co., Inc., South San Francisco, CA; Merck & Co., Inc., South San Francisco, CA; Merck & Co., Inc., South San Francisco, CA; Merck & Co., Inc., South San Francisco, CA; Celerion, Lincoln, Nebraska; Merck & Co., Inc., South San Francisco, CA; Merck & Co., Inc., South San Francisco, CA

## Abstract

**Background:**

Ulonivirine (ULO; MK-8507), a nonnucleoside reverse transcriptase inhibitor, is in development with islatravir as an oral weekly HIV-1 treatment. Rifabutin is a moderate inducer of CYP3A4; thus, coadministration with ULO could potentially lead to decreased ULO exposure. This Phase 1 study was conducted to investigate the effects of rifabutin on the pharmacokinetics (PK) of ULO.

**Figure:**
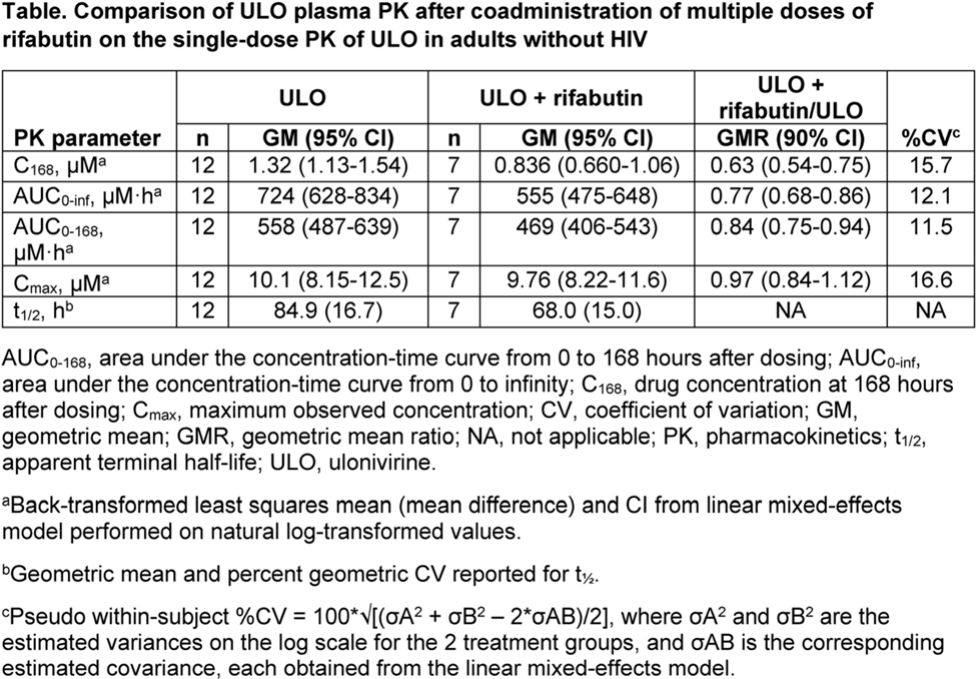

**Methods:**

The interaction between ULO and rifabutin was assessed in an open-label, 2-period, fixed-sequence study in adults without HIV. In Period 1, a single dose of ULO 400 mg was given on Day 1. In Period 2, rifabutin 300 mg was given daily for 27 days, and, on Day 14, participants received a single dose of ULO 400 mg. Samples were collected for plasma PK and urine M9 metabolite analysis. To assess the interaction between ULO and rifabutin, the ratio of ULO concentrations (C_168_) with and without coadministration of rifabutin were calculated. The interaction was considered not clinically meaningful if the lower bound of 90% CI of the true geometric mean ratio (GMR; ULO + rifabutin/ULO) for C_168_ was above 0.5. Safety was monitored by clinical (physical examination, vital signs, electrocardiograms) and laboratory assessments.

**Results:**

Twelve participants (8 males, 5 White) were enrolled; median age was 39.5 years (range 20-65). When ULO was coadministered with multiple doses of rifabutin, t_1/2_ decreased from 84.9 hours for ULO alone to 68.0 hours for ULO + rifabutin (Table). The GMRs (90% CI) for C_168_ and area under the concentration-time curve from time 0 to infinity (AUC_0-inf_) were 0.63 (0.54-0.75) and 0.77 (0.68-0.86), respectively. Six participants discontinued due to adverse events (AEs) related to rifabutin (n=5; leukopenia [n=4], thrombocytopenia [n=2], fever [n=1]) or ULO + rifabutin (n=1; myalgia). Two participants who received ULO alone experienced AEs considered related to ULO (upper abdominal pain and constipation). The most frequently reported AE for participants who received ULO + rifabutin was headache (n=3), considered related to study intervention. There were no clinically meaningful changes in other safety assessments.

**Conclusion:**

Rifabutin modestly reduced ULO exposure, but this was not considered therapeutically relevant. ULO 400 mg given alone or with rifabutin was generally well tolerated.

**Disclosures:**

Makeda Robinson, MD, PhD, Merck & Co., Inc: Employment|Merck & Co., Inc: Stocks/Bonds (Public Company) Randolph P. Matthews, MD, PhD, Merck & Co., Inc.: Employee|Merck & Co., Inc.: Stocks/Bonds (Public Company) Bhargava Kandala, PhD, Merck & Co.: Employment|Merck & Co.: Stocks/Bonds (Public Company) Akshita Chawla, PhD, Merck & Co., Inc: Employment|Merck & Co., Inc: Stocks/Bonds (Public Company) Arinjita Bhattacharyya, PhD, Merck & Co., Inc: Employment|Merck & Co., Inc: Stocks/Bonds (Public Company) Carol Cannon, MSN, Merck & Co., Inc: Employment|Merck & Co., Inc: Stocks/Bonds (Public Company) Andrea Schaeffer, MS, Merck & Co., Inc: Employment|Merck & Co., Inc: Stocks/Bonds (Public Company) S. Aubrey Stoch, MD, Merck & Co., Inc: Employment|Merck & Co., Inc: Stocks/Bonds (Public Company) Jesse C. Nussbaum, MD, Merck & Co.: Employee|Merck & Co.: Stocks/Bonds (Public Company)

